# Impact of agriculture and land use on nitrate contamination in groundwater and running waters in central-west Poland

**DOI:** 10.1007/s10661-016-5167-9

**Published:** 2016-02-17

**Authors:** Agnieszka Ewa Lawniczak, Janina Zbierska, Bogumił Nowak, Krzysztof Achtenberg, Artur Grześkowiak, Krzysztof Kanas

**Affiliations:** Department of Ecology and Environmental Protection, Poznan University of Life Sciences, 94C Piątkowska Street, 60-649 Poznan, Poland; Institute of Meteorology and Water Management, National Research Institute, 174/176 Dąbrowskiego Street, 60-594 Poznań, Poland; Environment and Water Consulting, 165 Piątkowska Street, 60-650 Poznan, Poland; Wielkopolska National Park, Jeziory, 62-050 Mosina, Poland

**Keywords:** Fertilization, Land use, Nitrate Directive, Protected area

## Abstract

Protected areas due to their long-term protection are expected to be characterized by good water quality. However, in catchments where arable fields dominate, the impact of agriculture on water pollution is still problematic. In Poland, recently, the fertilization level has decreased, mostly for economic reasons. However, this applies primarily to phosphorus and potassium. In order to evaluate the impact of agriculture on water quality in a protected area with a high proportion of arable fields in the aspect of level and type of fertilization, complex monitoring has been applied. The present study was carried out in Wielkopolska National Park and its buffer zone, which are protected under Natura 2000 as Special Areas of Conservation and Special Protection Areas. The aim of the study were (1) to assess the impact of agriculture, with special attention on fertilization, on groundwater, and running water quality and (2) to designate priority areas for implementing nitrogen reduction measures in special attention on protected areas. In our study, high nitrogen concentrations in groundwater and surface waters were detected in the agricultural catchments. The results demonstrate that in the watersheds dominated by arable fields, high nitrogen concentrations in groundwater were measured in comparison to forestry catchments, where high ammonium concentrations were observed. The highest nitrogen concentrations were noted in spring after winter freezing, with a small cover of vegetation, and in the areas with a high level of nitrogen application. In the studied areas, both in the park and its buffer zone, unfavorable N:P and N:K ratios in supplied nutrients were detected. Severe shortage of phosphorus and potassium in applied fertilizers is one of the major factors causing leaching of nitrogen due to limited possibilities of its consumption by plants.

## Introduction

Eutrophication is a key factor causing degradation of water quality, which restricts its use. Recently, agriculture is recognized as a major source of water pollution, which is also the most difficult to eliminate due to its spatial character (Billen et al. 2013; Fowler et al. 2013). Degradation of soil and water from agriculture occur due to residues of used chemicals (pesticides), emission of ammonium, methane or sulfide from livestock production, and livestock manures. One of the most problematic is nitrogen and phosphorus leaching from arable fields to groundwater and surface water as a result of higher amounts of these nutrients applied in natural and mineral fertilizers compared to plant requirements or supplied in adverse conditions (Billen et al. 2013; Kyllmar et al. 2014b).

The major excessive N inputs from agriculture have been identified as a major contributor to stream N loadings (Boyer et al. 2002; Hatano et al. 2005; Garnier et al. 2010). Nutrient leaching depends on several factors, primarily fertilization level, type, and timing of fertilizer application; the method of their application to the soil; properties of soils (i.e., pH, structure and organic matter content), types of crops and their fertilizer requirements; method of cultivation and agronomic practices; and the level of animal production (Bechmann 2014; Kyllmar et al. 2014a, 2014b). Weather conditions and catchment land use also have a crucial impact on the intensity and quantity of nitrogen leaching (Jiang et al. 2014; Yoon 2005; Woli et al. 2008).

A key factor determining plant nutrient uptake is also the availability of microelement and macroelement in the soil, particularly mass ratios between elements (Cakmak 2005; Fageria 2001; Güsewell et al. 2003; Szczepaniak et al. 2013). As shown in the study of Lawniczak et al. (2009) and Lawniczak (2011), an insufficient amount of potassium reduces nitrogen uptake by plants and thereby may increase nitrogen leaching from soil. Also, deficient availability of phosphorus causes decreased plant biomass, even when nitrogen is in an optimal concentration compared to plant requirements (Güsewell 2004). However, relationships between these elements are not well understood in terms of nutrient leaching in agricultural areas.

Nitrogen, in particular the very soluble nitrate, is easily dissolved into the percolating water. Phosphorus is less mobile and reaches surface water due to erosion with the bound soil particles. These different pathways cause a problem with water protection, because elimination of one water pollution source may aggravate another. For example, reduction of fertilization level or one of the elements may not reduce leaching of nutrients as a result of the unfavorable ratio of nutrients in soil. Deficiency of phosphorus or potassium limits the uptake of nitrogen by plants, even when the nitrogen level is sufficient (Lawniczak et al. 2009). This suggests that at a low level of fertilization due to shortage of potassium and phosphorus, there may occur loss of nitrogen, which results in water and soil pollution. This issue may concern two thirds of the world’s agricultural land where potassium deficiency occurs (Römheld and Kirkby 2010).

The necessity of measures to reduce the negative impact of agriculture on water quality results from the provisions of European Commission Council Directive 91/676/EEC (i.e., the Nitrate Directive) concerning the protection of waters against pollution caused by nitrates from agricultural sources. In Poland, these activities are obligated in the designated Nitrate Vulnerable Zones (NVZs), which were introduced as special actions based on local law. However, nitrogen pollutants affect more areas (Iital et al. 2014; Rozemeijer et al. 2014; Wendland et al. 2009), even where the nitrate level is exceeded occasionally. Particularly, they should be focused on protected areas that are characterized by a large proportion of agricultural land.

The Wielkopolska region is one of the most developed agricultural areas in Poland. A high proportion of agricultural land cover types in the region carry the risk of water pollution. The fertilization level in this part of Poland was always higher than in other parts of Poland (GUS 1952–2013). However, recently, these differences significantly decreased and application of fertilizers is at the level recommended in terms of water and soil protection against pollution from agricultural sources (Codex of Good Agricultural Practice 2004).

In order to recognize the impact of agriculture, particularly supply of fertilizers, on water quality in the protected area, complex monitoring has been applied. The study was carried out in Wielkopolska National Park and its buffer zone, which are also protected as Natura 2000 sites. In this area, open water bodies are characterized by poor water quality (Lawniczak unpublished results). Knowledge of the impact of agriculture on groundwater quality, particularly the most problematic non-point sources, is crucial for a proper protection strategy for this area.

The aim of the study were (1) to assess the impact of agriculture, particularly fertilization, on quality of groundwater and running water; (2) to evaluate the effect of fertilization level on nitrogen concentrations in the protected area and its buffer zone; and (3) to designate priority areas for implementing nitrogen reduction measures.

## Study site

The study was carried out in the Wielkopolska National Park (Wielkopolski Park Narodowy, WPN) and its buffer zone, located in the lowland, central part of Poland, about 15 km from Poznań City, on the left bank of the Warta River. The park was established in 1957, on an area of 5244 ha. In 1996, the buffer zone of the park was established in order to reduce the human impact on the park and improve the efficiency of its protection. However, restrictions in the buffer zone are less strict than in the park. Currently, the park covers 7597.20 ha and its buffer zone 7242.8 ha (Cykowiak 2013). The majority of the park has been protected since 1934. In 2001, this area also became protected within the Natura 2000 European Network, called Ostoja Wielkopolska, which is a Special Area of Conservation (SAC) under the Habitats Directive, and since 2003 as a Special Protection Areas (SPAs), under the Birds Directive called Ostoja Rogalińska. The area of the park and its buffer zone are spread between the following five communes: Stęszew, Mosina, Puszczykowo, Dopiewo, and Komorniki.

**Fig. 1:**
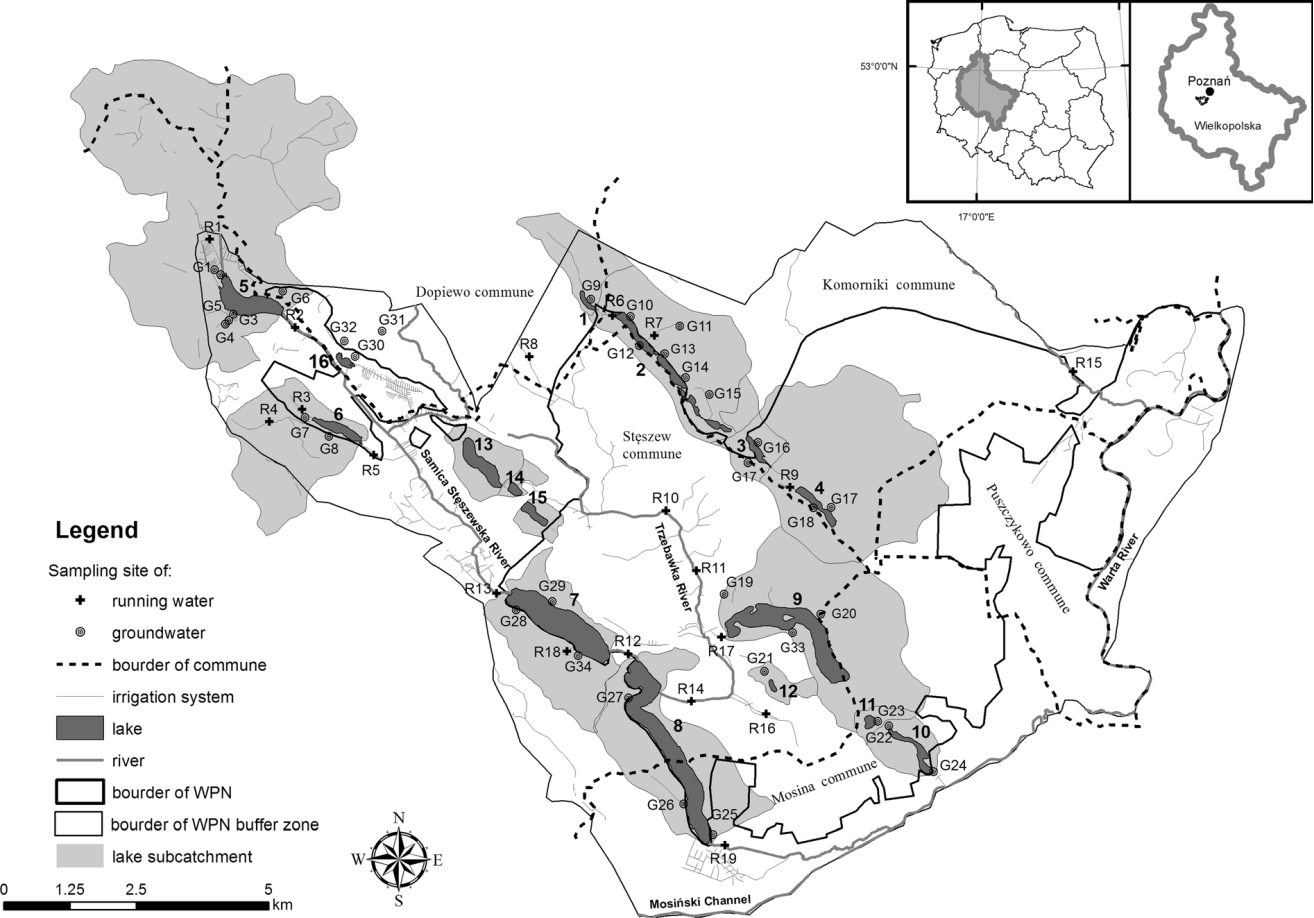
Location of the sampling sites of groundwater and surface water in the studied catchments. Abbreviations: *1* Konarzewskie, *2* Chomęcicko_Rosnowskie, *3* Małe, *4* Jarosławieckie, *5* Tomickie, *6* Wielkowiejskie, *7* Witobelskie, *8* Łódzko-Dymaczeswskie, *9* Goreckie, *10* Budzyńskie, *11* Kociołek, *12* Skrzynka, *13* Dębno, *14* Bochenek, *15* Lipno, *16* Trzcielińskie lakes

Water bodies are located in two major river valleys (Fig. 1). The biggest is the trough of Samica Stęszewska River. The river connects the following four lakes: Niepruszewskie, Tomickie, Witobelskie, and Łódzko-Dymaczewskie. The Tomickie Lake is located in the buffer zone of the WPN, while Witobelskie and Łódzko-Dymaczewskie Lakes are in the park. Between Tomickie and Witobelskie Lakes, the river is fed by two ditches from the Trzcielińskie and Wielkowiejskie catchments. However, Trzcielińskie Lake is characterized by pond features (small area, depth of 0.3 m, and thick bottom sediments) due to high disappearance rate. For the present analysis, except groundwater measurements, this lake was not included. The second and biggest trough in WPN is Konarzewsko-Chmęcicko-Rosnowskie. Konarzewskie Lake, located in the buffer zone of Wielkopolski National Park, is connected to Chmęcicko-Rosnowskie Lake by a ditch. The remaining two lakes in this valley (Lakes Małe and Jarosławieckie) are not trough flow lakes. The last complexes of lakes are located in the southern-east part of the WPN (Góreckie-Budzyńskie gutter, which contains Lakes Góreckie, Skrzynka, Kociołek, and Budzyńskie) and in the buffer zone (Dębno, Bochenek, and Lipno). In the park, there are 11 lakes, and 5 in the buffer zone, the area of which varies from 1.7 ha for Skrzynka Lake to 127.0 ha for Łódzko-Dymaczewskie Lake. The ecological status of the lakes is not satisfactory. Nine lakes of 15 are characterized by moderate status, 2 poor, and 4 bad, evaluated on the basis of biological, physico-chemical, and hydromorphological parameters (Lawniczak et al. 2013). The lake catchments vary from 0.34 km^2^ for Kociołek to 9.14 km^2^ for Jarosławieckie Lake (Table 1).

**Table 1:** Lake surface, catchment areas, and land use in the studied catchments

Lake	Lake surface	Direct catchment area	Total catchment area	Urban areas	Grasslands and pasture	Forest	Wetlands	Arable fields	Fallow lands	Gardens	Other waters
	ha	ha	km^2^	Percent of direct catchment area							
Konarzewskie	2.5	168.31	1.68	8.27	4.64	5.65	5.01	76.43	–	–	–
Chomęcicko-Rosnowskie	41.2	465.27	6.36	10.16	1.00	14.95	2.09	70.18	–	1.54	0.08
Małe	6.6	82.28	0.82	15.66	–	13.90	0.31	53.24	6.02	10.87	–
Jarosławieckie	12.7	914.24	9.14	0.39	0.63	65.51	0.14	32.83	0.35	–	0.15
Tomickie	38.7	1847.51	69.05	7.78	0.70	30.96	2.38	57.13	0.30	0.13	0.62
Wielkowiejskie	15.4	428.99	4.29	3.66	0.62	16.99	2.61	73.51	1.67	0.57	0.37
Witobelskie	97.5	337.96	73.88	10.61	0.58	3.53	1.81	82.94	–	–	0.53
Łódzko-Dymaczewskie	127	445.05	110.16	12.15	2.08	42.08	–	43.44	–	–	0.25
Budzyńskie	13.5	96.9	0.97	5.53	–	79.41	3.19	8.53	3.34	–	–
Góreckie	99.8	666.48	6.66	0.58	–	77.13	0.10	22.12	–	–	0.07
Kociołek	4.2	34.07	0.34	0.73	–	97.70	–	1.24	0.33	–	–
Skrzynka	1.7	48.37	0.48	0.94	–	87.99	4.61	6.46	–	–	–
Lipno	7.8	37.50	0.38	37.85	–	42.64	–	19.51	–	–	–
Dębno	19.0	100.57	1.01	44.49	–	12.35	4.18	32.08	2.53	4.18	0.19
Bochenek	3.0	25.02	0.25	35.02	–	1.63	18.70	28.82	9.44	6.21	0.18

Agricultural value of soils according to the soil valuation classes (Polish Soil Classification 2011) in the studied area is moderate and poor. The predominant soils are average quality (classes IIIb and IVa). In the northern part of the park, similar proportions of medium and poor ones (grades IIIb to V) are observed. Only in the southern part are poor and very poor quality soils prevalent (classes V and VI; GUS 2010). These poor soil types are confirmed by the classification of soil suitability, understood as agricultural usefulness. In the studied area, the predominant agricultural soil suitability complex is very good rye complex (good, compact soil structure; mainly heavy loamy sands or loamy sands), slightly less good rye complex (mainly light loamy sands; susceptible to drought, acidification, and leaching from the soil profile), and weak rye (mainly slightly deep loamy sands; low water capacity). In the southern part, mainly weak rye complex and very weak rye (the poorest soils; mostly composed of loose sands) dominate (GUS 2010).

Crop production is dominated by cereals, whose proportion in the sown area in the analyzed watersheds averaged 63.4 % and was slightly lower than in the Poznań district and Wielkopolska region. In the central-western part of the park (Stęszew commune), the cultivation of cereals is lower (55.0 %) than in the southern part (68.8 %). Unfavorable for water quality is the high proportion of maize cropping in the park and its buffer zone, which is significantly higher than in Poznan province and Wielkopolska region and varies from 6.3 % of area in the western part (Stęszew commune) of the park to 11.5 % in the north-western part (Dopiewo commune). Wide inter-rows, delayed growth of plants at the beginning of the growing season and high fertilization cause higher probability of nutrient leaching from field corn production than cereal fields (Zbierska et al. 2002).

Within animal production, the most popular is pig farming, which was maintained in 44.6 % of farms in the middle and western parts of the WPN and its buffer zone (Stęszew district), in 32.5 % of farms in the north-western part (municipality of Dopiewo), in 17.0 % of farms in the south-eastern part (Mosina district), and only in 7.9 % of farms in the northern part (Komorniki commune; GUS 2010). Cattle farms specialize mostly in the middle and western parts of the park (24.3 % of farms in the municipality of Stęszew), slightly less in the eastern part (13.5 %), and least in the northern part (5.4 %). There is a high proportion of poultry farming in the south-western and north-western parts of the park and its buffer zone, primarily chicken breeding (32.5 and 19 % of the farms, respectively).

## Methods

The study was carried out in the 15 catchments of the lakes located in the Wielkopolska National Park (WPN) and its buffer zone. In order to evaluate the impact of agriculture on water quality of these lakes, a comprehensive study of water quality—groundwater and running water supplying lakes—and a questionnaire survey including level of fertilization and type of production of the arable fields in the analyzed catchments were carried out. The field survey was completed in 2012.

### Catchment land cover

Drainage boundaries were determined from a topographic map for a strategy protection plan for WPN (Lawniczak et al. 2013). The total and sub-catchment of each lake were assigned. The sub-catchment (i.e., direct catchment) covers the watershed area of the catchment with surface runoff flowing directly into the lake. The total catchment contains the drainage area of land where surface water converges directly and indirectly (for example, through lakes and streams) to the analyzed lakes. Land use of the catchment of each lake located in the WPN and its buffer zone was determined based on the orthophotomaps made in 2011 (pixel 10 m; source WPN) and field observation.

Creation of a vector map of land use involves digitizing the orthophotomap and broadcasting individual descriptive attributes of the area resulting from the visual analysis of the orthophotomap. This work was carried out in ArcGIS in the 1992 National Geodetic Coordinate System.

For the study, the following classification of management area was adopted:

- Urban areas (including residential buildings, industrial, and transport such as roads and railway);
- Meadows and pastures;
- Forest and woodlots;
- Wetlands;
- Arable fields;
- Parks and gardens (including communal, allotments, and cottage gardens);
- Fallow lands (wasteland)—areas uncultivated or barren and undeveloped areas in the vicinity of residential buildings whose function was difficult to qualify;
- Other waters—both natural and superficial water bodies.

### Groundwater and running water

**Fig. 2:**
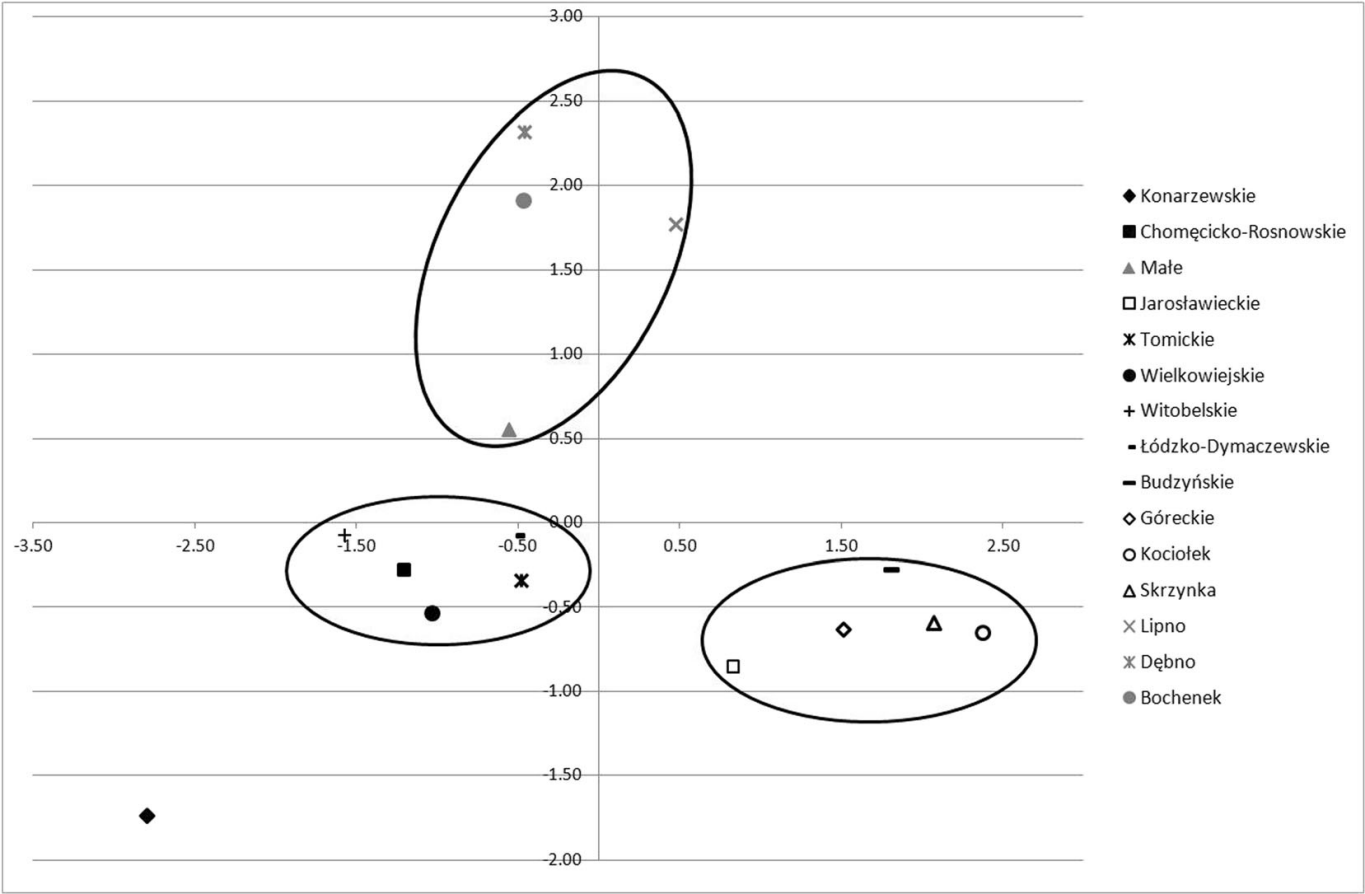
Distribution of the studied lakes in the system of principal component analysis (PCA), based on land use in the lakes’ sub-catchments

Groundwater samples were collected in the wells installed in each studied catchment, a short distance from each lake. In total, 34 wells were established (Fig. 1). The depth of each well was determined by groundwater water level. Water chemistry was analyzed in spring (April) and summer (June/August) 2012. Localization of the sampling sites is shown in Fig. 2.

Surface water quality from 19 sites from 14 different water courses supplying lakes located in the Wielkopolska National Park and its buffer zone was measured (Fig. 1). At Samica Stęszewka River, 6 sites were chosen (at inflow and outflow of lakes), 11 at different drainage ditches supplying water from watersheds, 4 sites at Trzebawka stream, and 1 at Wirenka River. Surface water in water courses was collected in spring (in April) and twice in summer (June and July/August) 2012.

Conductivity and pH in groundwater and running water were measured in situ using potentiometers (Elmetron CX-401, Elmetron CC-551, respectively). For soluble chemical parameters, samples were filtered using Sartorius Cellulose filters with nominal pore size of 0.45 μm. All samples were stored in the fridge and analyzed no later than 8 h after collection.

In freshwater, beside pH and conductivity, temperature and dissolved oxygen were measured in situ. In the laboratory of the Department of Ecology and Environmental Protection, Poznan University of Life Sciences, the following parameters were measured: soluble reactive phosphate (SRP; amino acid method), total phosphorus (TP; acid persulfate digestion method), nitrate (NO_3_^−^; cadmium reduction method), ammonium (NH_4_^+^; Nessler’s method), nitrite (NO_2_^−^; ferrous sulfate method), organic nitrogen (Norg; Kjeldahl’s method), and sulfur (SO_4_^2−^; colorimetric method), determined using a spectrophotometer (HACH DR/2800). Potassium (K^+^) concentrations were measured with flame emission spectroscopy, on a Shewood model 425. Chloride (Cl^−^), magnesium (Mg^2+^), and calcium were obtained with the titration method. Biochemical oxygen demand (BOD5) was measured using the Winkler method. Alkalinity was assessed with sulfuric acid to an end point of pH = 4.5 using a pH analyzer (Elmetron CPI-551).

### Agriculture

The level of fertilization in each catchment was determined on the basis of the field survey and statistical data obtained from the Central Statistical Office in Poland in relation to the communes which are located in the studied catchments. To improve knowledge of the type of agricultural activities in the park, a questionnaire survey was conducted among farmers leasing agricultural land under the management of the WPN. The questionnaire survey included information about conditions and the level of agricultural production (land use structure and the structure of crops, cultivation technologies, the level of fertilization and applied chemicals, crop yields and harvest crops, plowed products, remains of papilionaceous plants, and structure and density of livestock), sale of crops, and purchase of production resources. Personal contact with farmers allowed us to characterize agricultural production on the leased fields and draw up the balance of nutrients for 2 years, 2011 and 2012.

Nutrient balance of arable land was calculated in accordance with the Regulation of the Minister of Environment of 23 December 2002 on the detailed requirements to be met by the programs of measures aimed at reducing the outflow of nitrogen from agricultural sources (Regulation… 2002). Nitrogen balance (N, P_2_O_5_, and K_2_O) was calculated by the method called “on the field surface” according to the formula

$$ S=Y-X\left[\mathrm{kg}\ \mathrm{N}/\mathrm{ha}\right] $$

where

*S*: Balance account,
*Y*: Total inflow of nitrogen,
*X*: Total outflow of nitrogen.

Nitrogen inflow (*Y*) covers

$$ Y=A+B+C+D+E $$

where

*A*: Mineral fertilizers,
*B*: Natural fertilizers,
*C*: Ploughed by-products (i.e., postharvest residues straw and leaves),
*D*: The remains of papilionaceous plants,
*E*: Nitrogen in atmospheric precipitation.

The amount of the remains of papilionaceous plants was calculated based on the area of arable field covered by papilionaceous plants and the value of N fixation according to Nährstoffvergleich… (1997). Nitrogen in atmospheric precipitation in the Wielkopolska region from 2011 and 2012 was evaluated by the Voivodship Environmental Protection Inspectorate in Poznań (Environment 2012, 2013).

Nitrogen outflow (*X*) from fields covers

$$ X=F+G $$

where

*F*: Total crop production from arable land
*G*: Production from grasslands.

### Statistical analyses

Differences in water quality parameters measured in streams and groundwater within studied catchments were analyzed using repeat measurement ANOVA and a posteriori Tukey’s test. Principal component analysis (PCA) was employed to investigate relationships between water quality parameters from different catchments, without any a priori assumptions. The statistical analyses were performed using Statistica (StatSoft, Poland) software. To assess normality of variance, data were transformed (square). For the exploration of main gradients governing the water quality parameter distribution, detrended correspondence analysis (DCA) was applied (ter Braak and Šmilauer 1998). The relationship between water quality parameters and land use as well as level of fertilization in each studied catchment was analyzed based on redundancy analysis (RDA) with the Canoco program. To determine the most important variables, automatic forward selection of environmental parameters was used with the Monte Carlo perturbation test (with 456 perturbations) (Lepš and Šmilauer 2003).

## Results

### Land use

Within analyzed watersheds of lakes located in the Wielkopolska National Park and its buffer zone, PCA distinguished three groups, characterized by different proportions of land use in the catchment (Fig. 2). Only Lake Konarzewskie is discriminated from the others due to a high proportion of arable fields, i.e., 76.43 % (Table 1). Two major groups were separated based on domination of field and forest. Watersheds of the Góreckie, Skrzynka, Kociołek, Jarosławieckie, and Budzyńskie Lakes were characterized by a high proportion of forest (average 81.55 % of catchment area). In contrast, Witobelskie, Chomęcicko-Rosnowskie, Tomickie, Łódzko-Dymaczewskie, and Wielkowiejskie catchments are dominated by arable fields (average 65.44 %). The third group consists of Bochenek, Małe, Lipno, and Dębno catchments, with the highest proportion of built-up areas (average 33.26 %) within all analyzed watersheds. In the catchment of the Lipno Lake, a significantly bigger area is covered by forest and built-up area compared to other watersheds, which amount to 42.64 and 37.85 %, respectively. In the Dębno watershed, built-up area covered 44.49 % with high proportion of arable fields, i.e., 32.08 %.

#### Fertilization and crop production

**Fig. 3:**
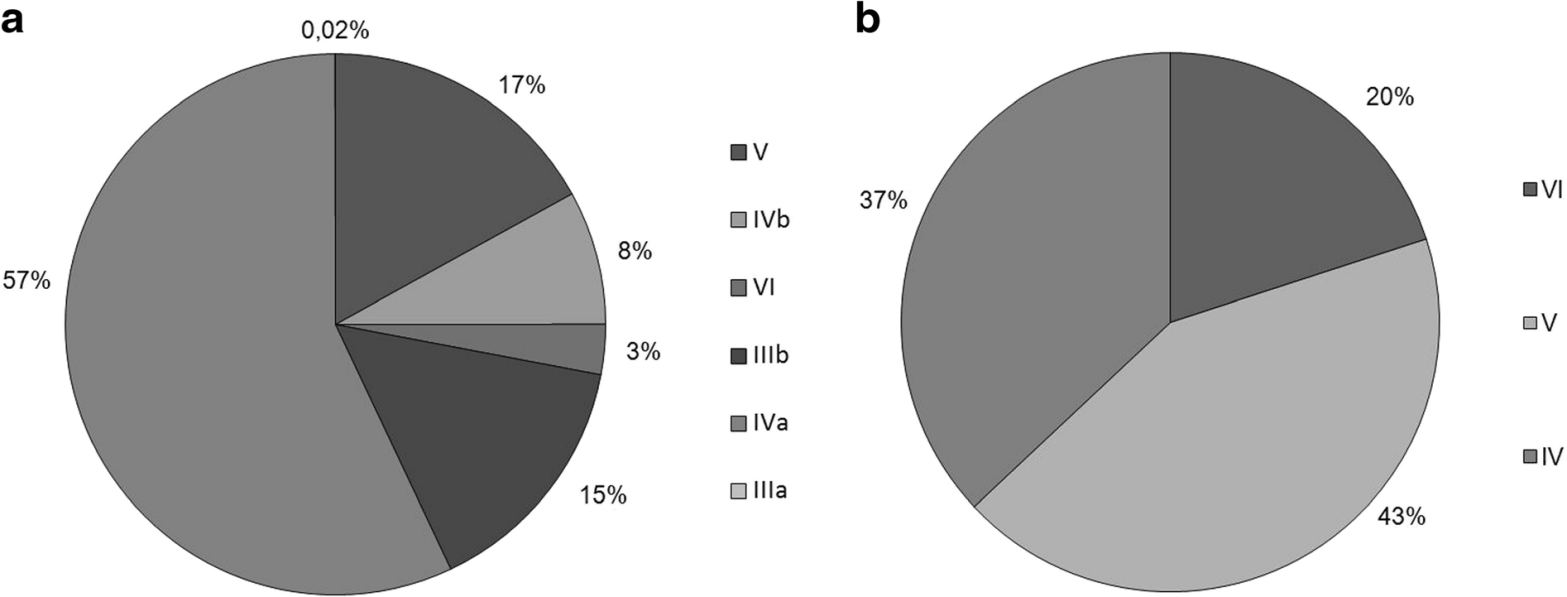
The proportion of the soil quality classes on leased **a** meadows and pastures and **b** arable land in the Wielkopolska National Park (Polish classification based on a scale from I to VI, where I is the best soil type and VI is the worst)

Agricultural area managed by direction of the Wielkopolska National Park amounts to 1333.8 ha, including 1192.6 ha of arable land and 141.2 ha of permanent grassland (meadows and pastures). Tenants of most of these lands are managed by individual farmers. Soils are mainly medium and low quality (Fig. 3).

Questionnaire and field surveys of arable land leased by farmers in the park showed very simplified crop structure. Between 2001 and 2012, the cultivated land was mainly basic cereals (Table 2). One reason for domination of cereal growing is less damage caused by wild animals than in other crops, particularly within fields located in the middle of the park among forest. A fairly large area was occupied by corn, mostly grain, and in 2012 also rape.

**Table 2:** The structure of sown arable land leased in the WPN in the years 2011 and 2012

Type of crop	2011	2012
	Percent	
Basic cereals	62.7	59.2
Winter rape	4.2	10.1
Sugar beet	5.1	6.7
Corn for grain	16.8	13.5
Corn on the green mass	2.5	2.1
Other crops	8.7	6.4

Analyses of the fertilization level in the buffer zone of WPN show quite high, except the southern part, level of mineral fertilization comparable with the average amount supplied in the district of Poznań and the Wielkopolska region (Table 3). The highest mineral fertilization occurs in the eastern part (Komorniki commune—Chomęcicko-Rosnowskie, Jarosławieckie, and Małe catchments), where annually, in total 199.1 kg NPK/ha is applied. In the Tomickie and Konarzewskie catchments, nutrient supply was slightly lower and amounted to 166.3 kg NPK/ha. In the central, western, and north-western parts of the park (Stęszew commune—Wielkowiejskie, Dębno, Bochenek, Lipno, Witobelskie, and Łódzko-Dymaczewskie catchments), fertilization averaged 149.7 kg NPK/ha, with a high proportion of nitrogen. In the southern part (Mosina commune—parts of Łódzko-Dymaczewskie and Góreckie catchments), fertilization was the lowest and was only 61.2 kg NPK/ha.

**Table 3:** Amount of fertilization used in the buffer zone of the WPN

Territorial unit	Fertilizer supply (kg/ha of farmland)					Supply nutrient ratio		
	Mineral (NPK)	N	P	K	Ca	N:P	N:K	P:K
Wielkopolska region	150.6	88.0	27.9	34.7	44.4	1:0.3	1:0.3	1:1.2
Poznań district	163.5	97.3	28.6	37.5	54.8	1:0.3	1:0.3	1:1.3
Dopiewo commune	166.3	109.3	25.7	31.3	53.4	1:0.4	1:0.3	1:1.2
Komorniki commune	199.1	94.8	41.5	62.8	102.3	1:0.2	1:0.2	1:1.5
Mosina commune	61.2	43.3	5.6	12.4	10.0	1:0.8	1:0.3	1:2.2
Stęszew commune	149.7	93.1	21.2	35.4	61.4	1:0.4	1:0.3	1:1.7
Average	117.0	68.8	19.0	29.2	45.4	1:0.4	1:0.2	1:1.5

In the central part of the park, analyses of the leased land showed a low fertilization level of arable fields (Table 4), where only a few farmers used organic manure and mineral fertilization averaged annually 85–86 kg nitrogen per hectare and 27–30 and 48–55 kg of phosphorus and potassium, respectively. However, there was wide variation of fertilizers used by individual farmers. In particular farms, nitrogen ranged from 59 to 129 kg/ha, phosphorus from 12 to 44 kg/ha, and potassium from 18 to 75 kg/ha. All farmers indicated that plowed by-products in the leased lands were absent. The balance of nutrients in 2011 showed an excess of nitrogen and a slight surplus of phosphorus and potassium. In 2012, a shortage of phosphorus and potassium in the arable fields located in the park was recorded (Table 4).

**Table 4:** Consumption and balance of nutrients on arable land leased in the WPN in the years 2011 and 2012

Specification	Use of natural fertilizers			Use of mineral fertilizers			Output of nutrients			Balance of nutrients		
	N	P_2_O_5_	K_2_O	N	P_2_O_5_	K_2_O	N	P_2_O_5_	K_2_O	N	P_2_O_5_	K_2_O
(kg ha^−1^)												
2011												
Mean	3	1	4	86	30	55	58	27	44	46	0.9	3.2
Range	3–25	1–13	6–30	66–116	12–44	18–75	21–105	10–39	14–68	−8–77	−29–26	−45–45
2012												
Mean	10	7	9	85	27	48	87	41	79	27	−9.2	−21
Range	0–35	0–32	0–35	59–129	12–42	18–70	32–121	24–54	47–106	−8–57	−34–33	−65–15

### Groundwater quality assessment

**Fig. 4:**
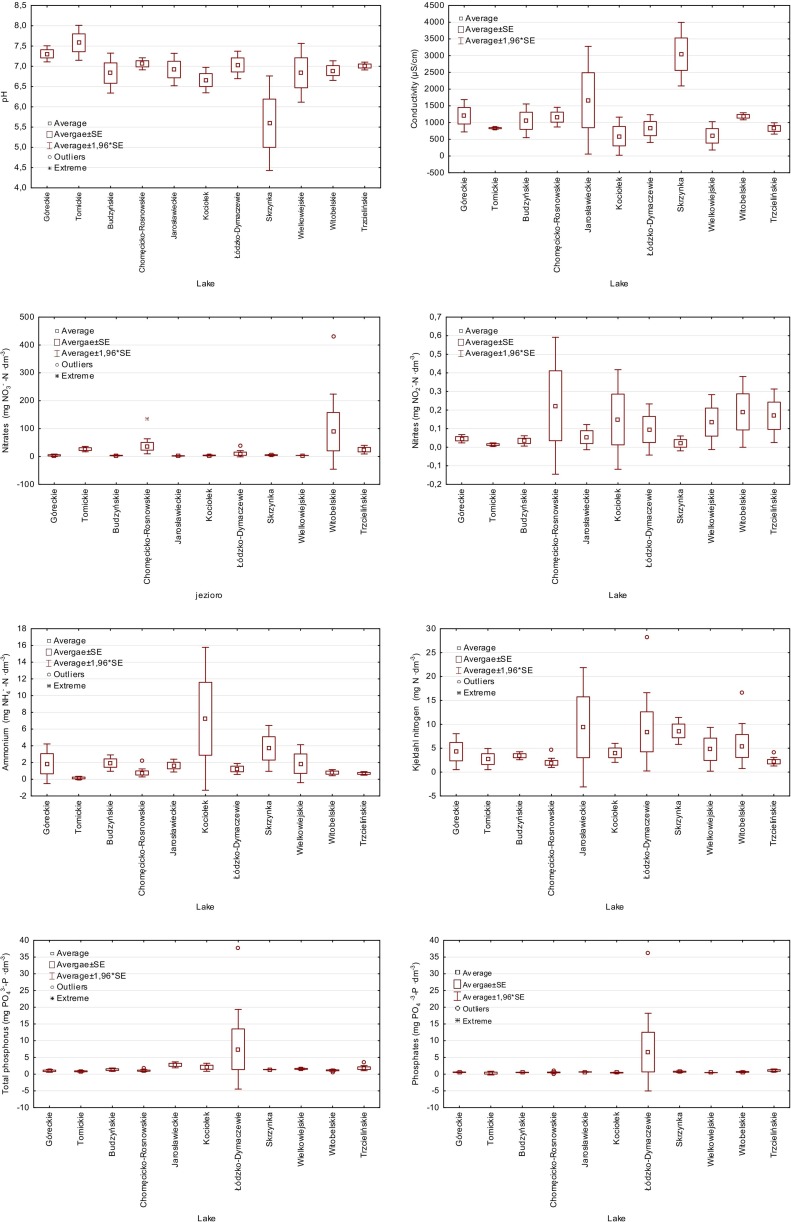
Variability of the selected physico-chemical parameters of groundwater in Wielkopolska National Park and its buffer zone

The groundwater measurements revealed high variability in nutrient concentrations within studied wells. Statistically significant differences between catchments were observed with respect to pH, conductivity, sulfur, chlorides, calcium, magnesium, hardness, and ammonium (Table 5). The lowest pH was detected in the forested Skrzynka catchment, which varied from 5.00 to 6.19. Remaining wells were characterized by neutral pH (Fig. 4). The highest concentrations of mineral contaminations were detected in the groundwater of Jarosławieckie and Skrzynka catchments, which achieved even 4130 and 3580 μS · cm^−1^, respectively. These values were mostly determined by high concentrations of calcium or chloride. The lowest conductivity was measured in Łódzko-Dymaczewskie catchment (292 μS · cm^−1^).

**Table 5:** Analyses of differences between studied groundwater quality based on physico-chemical groundwater parameters

Parameters	*df*	SS	MS	*F* value	*P* value
pH	10	5.419	0.542	4.48	**<0.001**
Conductivity	10	1752.071	175.207	2.75	**0.012**
Hardness	10	25.707	2.571	4.30	**0.001**
Magnesium	10	176.503	17.650	3.44	**0.003**
Calcium	10	269.959	26.996	2.42	**0.025**
Chloride	10	488.160	48.816	4.60	**<0.001**
Sulfur	10	573.731	57.373	2.15	**0.046**
Total nitrogen	10	16.039	1.604	0.75	0.676
Kjeldahl nitrogen	10	11.451	1.145	1.15	0.356
Ammonium	10	8.676	0.868	4.08	**0.001**
Nitrate	10	155.155	15.516	1.39	0.226
Nitrite	10	0.282	0.028	0.44	0.918
Total phosphorus	10	5.275	0.527	0.83	0.602
Phosphates	10	4.538	0.454	0.66	0.756
Alkalinity	10	287.354	28.735	1.23	0.308

All parameters except pH were square transformed. Significant differences are marked in bold (*p* > 0.05)

*df* degrees of freedom, *SS* sums of squares, *MS* mean sum of squares, *F* distribution, *p* level of significance

In the watersheds where arable fields dominate during springtime, high nitrogen concentrations were measured. In the Chomęcicko-Rosnowskie catchment, these values (136.7 mg NO_3_ · dm^−3^) exceed almost three times the limit value indicated in the Nitrate Directive (50 mg NO_3_ · dm^−3^) for areas identified as vulnerable to pollution from agricultural sources. These high concentrations were observed in spring. During summertime, nitrogen concentrations were lower but still elevated (29.4–47.2 mg NO_3_ · dm^−3^). In the Witobelskie catchment, these values are almost nine times higher (430.8 mg NO_3_ · dm^−3^). These extremely high concentrations were noted during springtime in the wells located in the arable fields, in the east part of the lake catchment. In summer, nitrate concentrations were lower (50.1 mg NO_3_ · dm^−3^) but still above the limit value. In the rest of the studied wells, nitrogen concentrations were much lower and ranged between 38.1 mg NO_3_ · dm^−3^ in the catchment of Lake Tomickie to 4.1 mg NO_3_ · dm^−3^ in Jarosławieckie and Wielkowiejskie watersheds (Fig. 4).

Concentrations of phosphates and total phosphorus in groundwater samples varied within studied catchments (Fig. 4). Extremely high values of phosphates and total phosphorus (phosphates 36.20 mg PO_4_ · dm^−3^ and total phosphorus 37.80 mg PO_4_ · dm^−3^) were measured in the wells located in the southern-west part of Łódzko-Dymaczewskie catchment. High phosphorus contaminations were observed also in the Jarosławieckie, Witobelskie, Chomęcicko-Rosnowskie, and Łódzko-Dymaczewskie catchments. Although significant differences between analyzed catchments were not detected, high concentrations of these nutrients still indicate problems with point sources of pollution. This was confirmed by a microbiological survey carried out in the catchments (Lawniczak et al. 2013).

The worst water quality within studied parameters was detected with respect to ammonium. In almost 35 % of samples, ammonium varied from 1.55 to 11.59 mg NH_4_ · dm^−3^, classified in fourth and fifth classes according the Polish classification. The highest concentrations were detected in the forested Kociołek catchment (Fig. 4).

**Fig. 5:**
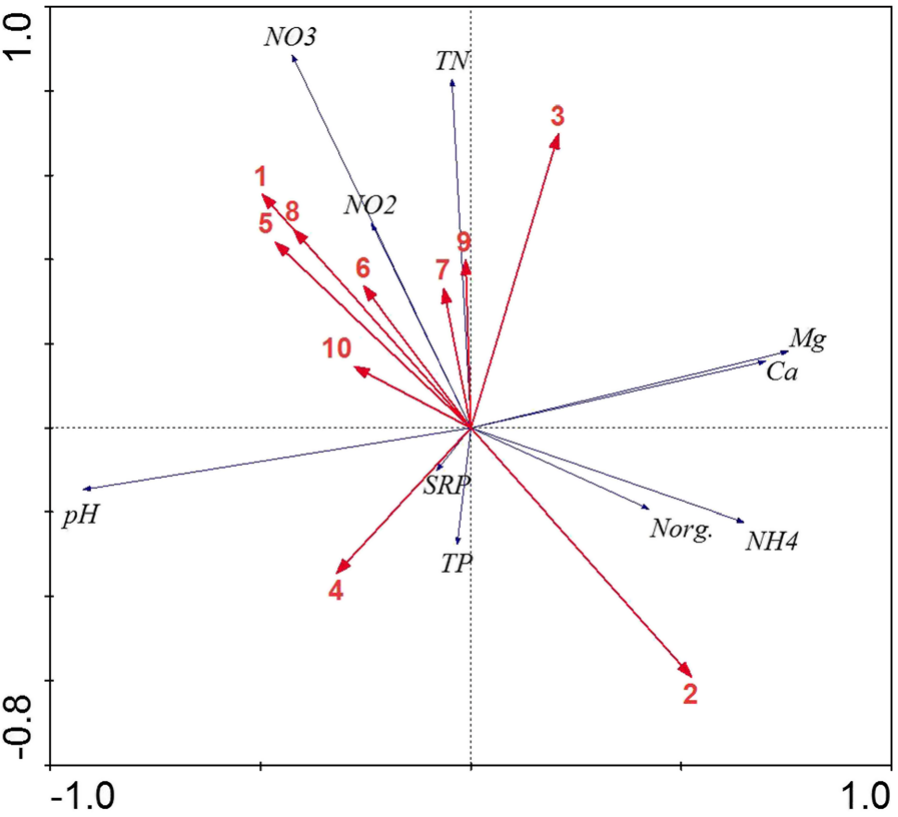
RDA ordination of the groundwater quality parameters and land use in catchments. Axis 1 explains 60.6 % of the water quality-land use relation and axis 2 explains 30.4 %. Abbreviations: *SRP* soluble reactive phosphate, *TP* total phosphorus, *NO*_*3*_ nitrate, *NO*_*2*_ nitrite, *NH*_*4*_ ammonium, *Norg*. organic nitrogen, *Mg* magnesium, *Ca* calcium, *1* arable fields, *2* forest and woodlots, *3* fallow lands, *4* meadows and pastures, *5* other waters, *6* parks and gardens, *7* wetlands, *8* urban areas, *9* N supply, and *10* P supply

All parameters which were selected as being influential on water quality parameters by the Monte Carlo procedure were used as input data for RDA analyses (Fig. 5). The first axis accounted for 60.6 % and the second axis for 30.4 % of the total variance in the relationships between groundwater quality and catchment parameters (type of land used in catchment and fertilization level). The values of arable fields, forest, grasslands, and N and P fertilizations were the parameters most associated with the values of groundwater quality parameters. The RDA results revealed arable fields as one of the most important parameters for nitrogen concentrations in groundwater and a high proportion of forest in the catchment for ammonium and organic nitrogen.

The relation between groundwater quality parameters, type of land use in catchment, and fertilization level was statistically significant (test of significance of first canonical axis *p* = 0.002, *F* ratio = 4.68 and test of all canonical axes *p* = 0.002, *F* ratio = 2.48).

### Running water quality assessment

The majority of the analyzed water courses supplying lakes located in the WPN were polluted by nitrogen as well as phosphates, total phosphorus, and Kjeldahl’s nitrogen (Table 6). High values of nitrogen in the small ditches supplying Chomęcicko-Rosnowskie, Wielkowiejskie, and Łódzko-Dymaczewskie lakes were detected and exceeded limit values indicated in the Nitrate Directive. The highest concentrations were measured in the ditches located in the agriculture parts of the catchments. In the Samica Stęszewska tributary, where nitrogen concentration was 302 mg NO_3_ · dm^−3^, cereal production dominated. In the Chomęcicko-Rosnowskie catchment, the highest nitrate concentrations were detected in the ditch located in the northern-east part of the lake, where maize cultivation dominated. This concentration varied from 0.83 in summer to 129 mg NO_3_ · dm^−3^ in spring. Also, in the major tributary supplying water to Chomęcicko-Rosnowskie Lake from Lake Konarzewskie, high total phosphorus and total nitrogen contents were recorded as well. Taking into consideration other measured parameters, the largest variations were found with respect to nitrite, chloride, and calcium within the analyzed catchment (Table 6). However, the correlation between analyzed water quality parameters, type of land use in catchment, and level of nutrient supply with fertilizer was not significant.

**Table 6:** Descriptive statistics of running water hydrochemistry

Parameter	Tomickie	Trzcielińskie	Witobelskie	Łódzko-Dymaczewskie	Wielkowiejskie	Chomęcicko-Rosnowskie	Lipno	Jarosławieckie
Hardness (mval · dm^−3^)	293.1 ± 0.0	439.38 ± 116.14	280.26 ± 127.87	359.03 ± 141.33	418.53 ± 3.571	495.95 ± 186.46	ND	ND
pH	7.7 ± 0.76	8.1 ± 0.24	7.40 ± 0.63	25.23 ± 37.59	7.94 ± 0.38	7.67 ± 0.49	7.96	7.27 ± 0.33
Conductivity (μS · cm^−1^)	738 ± 103	1011 ± 339	746 ± 97	771 ± 107	800 ± 87	1025 ± 241	922.00	676 ± 818
Alkalinity (mg CaCO_3_ · dm^−3^)	213.75 ± 37.12	251.25 ± 72.48	256.75 ± 52.32	213.44 ± 62.47	296.25 ± 68.94	236.67 ± 42.62	335.00	171.5 ± 19.09
COD (mg O_2_ · dm^−3^)	41.00 ± 2.55	29.35 ± 9.69	42.88 ± 19.55	46.49 ± 26.28	23.20 ± 2.12	30.88 ± 15.86	16.9	34.3 ± 3.11
Phosphates (mg PO_4_^3−^-P · dm^−3^)	0.40 ± 0.06	0.61 ± 0.28	0.49 ± 0.41	0.75 ± 0.68	0.49 ± 0.25	0.63 ± 0.58	0.59	0.33 ± 0.21
Total phosphorus (mg PO_4_^3−^ -P · dm^−3^)	0.7 ± 0.03	0.81 ± 0.10	0.98 ± 0.65	1.16 ± 0.81	0.66 ± 0.11	0.94 ± 0.66	0.65	0.66 ± 0.18
Nitrites (mg NO_2_^−^-N · dm^−3^)	0.05 ± 0.04	0.15 ± 0.18	0.02 ± 0.01	0.07 ± 0.08	0.012 ± 0.01	0.22 ± 0.25	0.01	0.01 ± 0.00
Nitrates (mg NO_3_^−^-N · dm^−3^)	1.15 ± 0.92	36.95 ± 50.98	0.69 ± 0.46	4.63 ± 8.45	5.2 ± 2.69	8.14 ± 11.23	3.3	0.1 ± 0.14
Ammonium (mg NH_4_^−^-N · dm^−3^)	0.32 ± 0.29	0.93 ± 1.12	0.40 ± 0.32	0.42 ± 0.80	0.10 ± 0.03	0.26 ± 0.15	0.1	0.24 ± 0.09
Organic nitrogen (mg NH_4_^−^-N · dm^−3^)	1.66 ± 0.27	1.55 ± 1.74	3.08 ± 1.85	3.24 ± 2.11	2.64 ± 0.70	2.01 ± 0.92	0.99	1.99 ± 0.63
Kjeldahl nitrogen (mg N · dm^−3^)	1.97 ± 0.02	2.48 ± 0.62	3.48 ± 2.09	3.66 ± 2.66	2.74 ± 0.67	2.26 ± 0.99	1.09	2.23 ± 0.53
Total nitrogen (mg N · dm^−3^)	3.17 ± 0.86	40.69 ± 52.12	4.30 ± 2.05	8.38 ± 9.50	7.31 ± 2.46	10.85 ± 11.09	4.4	2.33 ± 0.40
Sulfates (mg SO_4_^2−^ · dm^−3^)	91 ± 7.07	108 ± 11.31	69.5 ± 26.53	70.31 ± 21.20	110.00 ± 31.11	94.00 ± 34.69	134.00	79.00 ± 1.41
Chlorides (mg Cl^−^ · dm^−3^)	33.35 ± 13.08	35.55 ± 18.88	33.48 ± 17.95	33.48 ± 17.18	35.85 ± 20.58	41.70 ± 22.50	45.2	123.2 ± 9.62
Calcium (mg Ca^2+^ · dm^−3^)	98.67 ± 22.25	140.14 ± 32.36	94.44 ± 15.91	91.03 ± 26.25	82.91 ± 12.18	133.23 ± 44.55	64.35	74.36 ± 10.11
Magnesium (mg Mg^+2^ · dm^−3^)	9.09 ± 10.41	21.65 ± 8.57	34.43 ± 26.44	24.60 ± 15.36	45.03 ± 2.46	36.77 ± 14.10	75.34	21.65 ± 2.45
Oxygen (mg O_2_ · dm^−3^)	8.25 ±	14.2 ± 6.51	5.91 ± 3.29	8.76 ± 5.49	9.42 ± 6.19	10.45 ± 7.50	9.76	4.8 ± 1.75

*ND* no data

## Discussion

The results of groundwater and surface water analyses conducted in the 15 lakes’ catchments show high nitrogen concentrations in 12 % of analyzed samples. The biggest N inputs to groundwater and surface waters have been identified from areas of intensive agriculture compared to other land use categories such as forest or urban area. Also, Hatano et al. (2005), Hayakawa et al. (2006), and Kaushal et al. (2011) monitored N leaching from agricultural land use in catchments. The obtained results show the influence of a high proportion of arable fields in catchments on nitrogen concentrations in groundwater (Spearman’s rank order correlation coefficient *r* = 0.68, *p* < 0.05), while ammonium correlated positively with forested watersheds (Spearman’s rank order correlation coefficient *r* = 0.72, *p* < 0.05). The highest nitrogen concentrations were measured during springtime in the groundwater and running water located in the agricultural area, particularly area specialized in maze cultivation. In contrast, high ammonium concentrations in water were measured in the forested catchments (Boyer et al. 2002; Galloway et al. 2003). By contrast, phosphorus concentrations were higher in the area which is the local residential region.

Fertilization level in the park was relatively low, except for private fields in one of the studied catchments (Witobelskie watershed). A very high value of nitrate concentration, exceeding by almost nine times the indicated values for areas susceptible to nitrogen contamination from agricultural sources (according to the Nitrate Directive), was detected in one well. This indicates occasional excess of the level of fertilization. Moreover, it is very dangerous due to close location of the sources of pollution next to the lake.

The majority of the calculated nitrogen balance showed a small surplus in the leased fields in WPN, an average of 27 kg N/ha in 2012 to 46 kg N/ha in 2011. There was a large variation, with the balance of the various fields from a deficit (−8 kg N/ha) to highly positive (57 and 77 kg N/ha). The Codex of Good Agricultural Practice (2004) recommends nitrogen excess to 30 kg/ha in order to ensure sufficient availability of these nutrients for plants. In 70 % of the studied farms, these values were exceeded in 2011 and 40 % in 2012. Taking into consideration optimal types of nitrogen balance based on nutrient availability and deficiency in the farm (Wrzaszcz 2009), in the park, there dominated a deficient one, characterized by a negative balance between nutrients input and removal, in the range between 55.9 and 65.9 kg/ha. Fields with excessive fertilization were found only in 40 % of farms in the park in 2011 and none in 2012. However, in the many studied farms, a shortage of nitrogen was detected (50 % in 2011 and 90 % in 2012) below optimal values (55.9 kg N/ha per year) indicated for Wielkopolska region. Maintaining deficiency of nitrogen for a long time may lead to a reduction of deficit in soil fertility in this area. Fertilizer applied to the field in excess of plant needs is the biggest cause of water pollution.

Analyses of the leased land showed a lower fertilization level of arable fields than is noted in the Wielkopolska region. Only a few farmers used organic fertilizers. This follows on the one hand from the fact that the majority of farms specialize in crop production (and thus do not have their own natural fertilizers). On the other hand, the short term of lease and the uncertainty of its continuation cause abandonment of natural fertilization.

Although in the Wielkopolska National Park the fertilization level is low, in the buffer zone, nutrients are supplied in higher amount. Based on data from the Central Statistical Office of Poland (GUS 2010), the highest fertilization is applied in the western and east-northern parts of the buffer zone, which amounted to 166 and 199 kg NPK/ha, respectively. These values are higher than the average level applied in the region or in Poland. Taking into consideration the type of arable area, this situation is not favorable in terms of nature conservation of the park. In the sub-catchment maze cultivation dominated, production of which is characterized by broad intervals between sowing rows and a long time of soil without vegetation at the beginning of the growing season, which promotes nutrient leaching. According to the review of Groenendijk et al. (2008), denitrification losses in the soil occur mainly in the root zone in conditions of low oxygen, high organic matter, and high moisture contents. In contrast in the aerated soils with low organic matter and low water contents, denitrification rates will be lower. Therefore, on soils with weak rye and very weak rye complex occurring in the park and in the fields with a small cover of vegetation, degradation of a certain amount of nitrogen to molecular nitrogen by denitrification processes might be low, which may cause a high risk of displacement of N surpluses to surface water.

Also, high nitrogen concentrations during wintertime when freezing-thawing cycles occur influence high N exports in the agriculture areas due to soil freezing and thawing cycles Jiang et al. (2014). The study was conducted in the spring preceded by winter freezing bereft of snow cover. According to Bullock et al. (1988), freezing and thawing causes destruction of soil aggregates and acceleration of soil crusting, with ensuing erosion resistance and decreased infiltration. Soil freezing changes hydrological pathways (McNamara et al. 2008), mostly during a winter with a lack of snow cover and snowmelt season. Also, N fertilizer application in late autumn or early winter, which was applied in the studied area, according Jiang et al. (2014) is more sensitive to higher N exports, particularly during the snowmelt season rather than a rainy period. All these factors combined with biological processes and low N uptake by plants would enhance N leaching from agricultural areas during early springtime, which is reflected in high nitrogen concentrations in the groundwater and running water.

Moreover, concentrations of phosphorus and potassium in groundwater and surface water were high, but in comparison to soil types, level of fertilization, and plant requirements, a severe shortage of phosphorus and potassium in the soil was noted. Deficiency of these elements may cause leaching of nitrogen due to limited possibilities of its consumption by plants. Fotyma and Kuś (2000) indicated that the P:K ratio calculated as the amount of nutrients in plant yield, depending on nutrient availability in the soil, should amount to 1:1.5 at very low contents, 1:1.25 at small, 1:1 at medium, 1:0.75 at large, and 1:0.5 at very high contents. In the soil of the Wielkopolski National Park, the P:K ratio was favorable. Strong deficiency of P and K indicate high N:K and N:P ratios. According to Grzebisz (2004), the N:K ratio in arable fields should be 1:1.2. Lawniczak (2011) and Lawniczak et al. (2009) for wetland species observed that the optimal N:K ratio for plant growth is 1.5. However, this study was tested on grass species under control conditions. Grzebisz and Diatta (2012) indicate that potassium deficiency is one of the most important limiting factors of plant productivity in Poland, often unappreciated by farmers.

In the studied area, there is a noteworthy unfavorable ratio of N:P:K of 1:0.4:0.2, due to the downturn in agriculture and farmers conserving funds for fertilizers. Farmers often refrain from phosphorus-potassium fertilizer, which gives lower yielding than the effect of nitrogen fertilization. This trend is observed not only in Wielkopolska region but also in Poland since 1990, when political changes occurred and an increase of fertilizer prices took place (Table 7). During the last 50 years, the N:K supply ratio decreased from 1:1.4 to 1:0.3 in the Wielkopolska region. This trend continues, also with respect to N:P fertilization. These results suggested that limited uptake of nitrogen by plants due to the phosphorus and/or potassium deficiency can cause nitrate leaching from soil. Potassium shortage is observed in many countries in Europe and Asia (Grzebisz and Diatta 2012; Römheld and Kirkby 2010).

**Table 7:** Nutrient ratios in mineral fertilizers used in Poland and Wielkopolska region in the years 1950–2013

Year	Poland			Wielkopolska region		
	N:P	N:K	P:K	N:P	N:K	P:K
1950/1951	1:1.1	1:1.8	1:1.6	1:0.8	1:1.4	1:1.9
1960/1961	1:0.8	1:1.1	1:1.5	1:0.8	1:1.4	1:1.8
1970/1971	1:0.8	1:1.4	1:1.8	1:0.7	1:1.2	1:1.7
1980/1981	1:0.7	1:1.1	1:1.6	1:0.7	1:1.5	1:2.0
1990/1991	1:0.6	1:0.8	1:1.5	1:0.7	1:1.2	1:1.8
2000/2001	1:0.4	1:0.4	1:1.3	1:0.3	1:0.4	1:1.4
2010/2011	1:0.4	1:0.4	1:1.1	1:0.4	1:0.4	1:1.2
2012/2013	1:0.3	1:0.3	1:1.1	1:0.4	1:0.3	1:0.7

Source: (GUS 1952, 1961, 1971, 1981, 1991, 2001, 2011, 2013)

Lack of buffer zones and control of agricultural activities in the protected area and particularly in the lakes’ catchments caused degradation of water quality. The level of fertilization decreased in the Wielkopolska region, but still, there is a problem with water quality and implementation of the Nitrate Directive and Water Framework Directive (European Commission 1991, 2000). Intensive agricultural production and concentration of livestock production have played the role in water pollution and eutrophication in the upper section of the river Samica Stęszewska and Lakes Niepruszewskie and Tomickie (Zbierska et al. 2002), located beyond the protected area. Strong pressure of agriculture on water quality, a high concentration of nitrate, and eutrophication of Lake Niepruszewskie were the reasons to specify in 2003 the source section of the Samica Stęszewska River as sensitive, Lake Niepruszewskie as threatened by eutrophication, and the catchment area of the river to the cross section at the outlet of Lake Niepruszewskie as particularly vulnerable to nitrate (OSN). Contaminants from the upper part of the river Samica Stęszewska were transferred to the inflow of water and were a significant burden for lakes located in the downstream part of the water course, including lakes of Wielkopolska National Park (Zbierska and Kupiec 2005). In 2004–2012, repair programs were carried out to reduce the amount of nitrogen from agricultural sources (Regulation… 2004; Regulation… 2008). Implemented measures (Kupiec et al. 2008) resulted in improvement of water quality in the rivers and lakes (Lawniczak et al. 2008). However, this undertaken action was implemented only in the upper part of the Samica Stęszewska catchment. High accumulation of sediments in the lakes caused them to become shallow and in consequence acceleration of lakes’ disappearance (Lawniczak et al. 2011).

Analyses of groundwater and running water show high values of nitrate concentrations due to agricultural activities. To limit the risk of lake pollution from agriculture, it is necessary to focus efforts on improving the balance of supplied nutrients in the fields and reduce and control the quantity of nitrogen applied to the soil with fertilizers. Surprisingly, the highest values of nitrogen pollution were measured in the field located in the WPN, in the Witobelskie catchment. Other parts of catchments with a high degree of contamination such as Chomęcicko-Rosnowskie and Tomickie Lakes were located in the buffer zone of the park. Our study indicates that measured action combined with long-term monitoring data should be applied for proper protection of Wielkopolski National Park, not only in the protected area and its buffer zone but also in the whole catchment.

## Conclusions

The research was conducted at 15 lake watersheds in the Wielkopolska National Park and its buffer zone to examine the impact of agriculture, particularly crop production, on groundwater, and running water quality. Evaluation of water quality was crucial to assess the source of nitrogen pollution of lakes, which are characterized by high water trophy. Nitrogen leaching was also controlled by land use. The results demonstrate that in the watersheds dominated by arable fields, high nitrogen concentrations in groundwater were measured in comparison to forestry catchments, where high ammonium concentrations were observed. The highest nitrogen concentrations were noted in springtime after winter freezing, with a small cover of vegetation, and in the areas with a higher level of fertilizer application. In the studied areas, both in the park and its buffer zones, unfavorable N:P and N:K ratios in supplied nutrients were detected. Shortage of phosphorus and potassium may be one of the major factors causing leaching of nitrogen due to limited possibilities of its consumption by plants.
